# Estimating Vocal Fold Contact Pressure from Raw Laryngeal High-Speed Videoendoscopy Using a Hertz Contact Model

**DOI:** 10.3390/app9112384

**Published:** 2019-06-11

**Authors:** Manuel E. Díaz-Cádiz, Sean D. Peterson, Gabriel E. Galindo, Víctor M. Espinoza, Mohsen Motie-Shirazi, Byron D. Erath, Matías Zañartu

**Affiliations:** 1 Speech, Language & Hearing Department at the Boston University College of Health & Rehabilitation, Sargent College, Boston, MA 02215, USA; 2 Department of Electronic Engineering at Universidad Técnica Federico Santa María, Valparaiso 2390123, Chile; 3 Department of Mechanical and Mechatronics Engineering, University of Waterloo, Waterloo, ON N2L 3G1, Canada; 4 Department of Sound at Universidad de Chile, Santiago 8340380, Chile; 5 Department of Mechanical Aeronautical Engineering, Clarkson University, Potsdam, NY 13699, USA

**Keywords:** biomechanical modeling, contact pressure, endoscopy, Hertz impact, high-speed video, laryngeal high-speed videoendoscopy, vocal folds, tissue modeling

## Abstract

The development of trauma-induced lesions of the vocal folds (VFs) has been linked to a high collision pressure on the VF surface. However, there are no direct methods for the clinical assessment of VF collision, thus limiting the objective assessment of these disorders. In this study, we develop a video processing technique to directly quantify the mechanical impact of the VFs using solely laryngeal kinematic data. The technique is based on an edge tracking framework that estimates the kinematic sequence of each VF edge with a Kalman filter approach and a Hertzian impact model to predict the contact force during the collision. The proposed formulation overcomes several limitations of prior efforts since it uses a more relevant VF contact geometry, it does not require calibrated physical dimensions, it is normalized by the tissue properties, and it applies a correction factor for using a superior view only. The proposed approach is validated against numerical models, silicone vocal fold models, and prior studies. A case study with high-speed videoendoscopy recordings provides initial insights between the sound pressure level and contact pressure. Thus, the proposed method has a high potential in clinical practice and could also be adapted to operate with laryngeal stroboscopic systems.

## Featured Application

A method to directly estimate the contact pressure of the vocal folds using uncalibrated laryngeal kinematic data is presented. The approach is promising in enhancing the objective assessment of vocal function in clinical settings, especially for studying same-subject variations.

## 1. Introduction

The etiology of trauma-induced vocal fold (VF) lesions, such as nodules and polyps, has been long associated with detrimental vocal patterns and compensatory behaviors that result in increased VF collision pressures [1–5]. Unfortunately, a direct in vivo quantification of VF collision pressure is not feasible in routine clinical examinations due to the invasive nature of the procedure, i.e., placing a pressure probe in the glottis constitutes a risk for the VF tissue [6–8]. Therefore, current objective assessments of VF collision in clinical environments relies on indirect methods (e.g., aerodynamic [4], acoustic [9], ambulatory [10], etc.) and does not take advantage of the most direct and common procedure to clinically assess VF function: Laryngeal endoscopy. At the same time, the recent development of laryngeal high-speed videoendoscopy (HSV), with frame rates above 4000 fps, has significantly improved the temporal resolution of this medical examination, thus allowing for an accurate visualization and quantification of vocal fold vibration patterns that are challenging to capture using standard stroboscopic imaging methods [11–14]. In spite of these benefits, the adoption of HSV into the clinical practice has been slow, in part because current analysis methods and tools to navigate the data are not yet sufficiently intuitive or useful to the clinician [11,12]. Thus, there is a clear need for enhanced analysis methods for laryngeal endoscopy, and given the high prevalence of nodules and polyps [15], the estimation of VF collision pressure directly from endoscopic observations is poised to have a strong impact in the clinical practice.

In spite of the significant interest for directly assessing VF collision, only a few studies have been able to gather contact pressure data in human subjects by placing pressure probes at the glottis [6–8]. Sensing contact pressure is a challenge in terms of probe size (flow and tissue interference), sensor bandwidth, risk to the VF tissue, and subject tolerance during the examination. Nevertheless, these pilot studies reported values for peak collision pressure of 0.5 to 3.0 kPa [7] in normal participants and 1 to 4 kPa in subjects with organic pathologies, wherein the values were larger near the lesions [6]. A stronger collision has been associated with an increased voice intensity [8]. These results are consistent with intraglottal pressures reported from excised canine hemilarynges [16–18], where it was observed that the impact produced sharp pressure pulses that were positively correlated with subglottal pressure and VF elongation.

In addition to clinical measures, numerical approaches have been used to investigate the role of collision pressures in the development of VF lesions. Using internal stress or ad hoc solutions for computing contact, finite element studies have shown that (1) the elastic forces within the VF tissue are responsible for the impact strength [19]; (2) there is a relationship between the subglottal pressure and peak collision [19,20]; and (3) VF impact causes a transient in the tissue stress that can be related to fatigue damage [20] and a reduction of hydration [21]. More recently, models of collision have incorporated descriptions of fluid displacement in the VF cover [22] and asymmetric conditions [23] that better represent the underlying physics; these studies yielded VF contact pressures in the same range as the experimental data.

Silicone VF replicas have also been used to study VF collision [24,25]. In these studies, a digital image correlation (DIC) approach and a Hertzian impact model [26,27] were utilized to quantify surface strain distributions on the superior VF surface and to obtain collision pressures in silicone models of the VFs. The Hertzian contact model has also been used to quantify collision forces in numerically reduced order models of the vocal folds [28,29]. The Hertz contact theory provides a framework for quantifying non-adhesive elastic contact mechanics in terms of effective contact surface and material deformation by means of an apparent penetration. The general method has been validated against nonlinear finite element models, where it has been shown that it is a valid approach at small deformations [30]. When studying VF contact with Hertz contact [24,25], the VF geometry has been assumed to conform to a spherical shape and the Hertz penetration depth was estimated from surface strain extrapolation for known synthetic materials. The resulting contact pressure yielded results comparable to prior experimental studies [8,16]. It is worth noting that DIC is currently not feasible in vivo, since there is no safe way to apply a speckle pattern to the VF tissue. In addition and up to this point, the Hertzian approach required a calibration stage for the laryngeal HSV to compute the exact contact surface and apparent penetration in physical units. Raw HSV recordings can only provide relative VF dimensions. For the in vivo experiments, this problem is solved by projecting an additional laser grid on the glottis that is used to determine the scale with offline processing. However, the projected grid can introduce problems in the lighting conditions, can affect the temporal resolution as the grid can be difficult to observe at high frame rates, and requires custom made hardware [13,14,31,32]. Note that Hertz contact pressure also requires knowledge (or estimation) of VF tissue properties that are difficult, if not impossible, to directly measure in vivo.

Despite the simplicity and apparent validity of the Hertzian approach for estimating VF contact pressure in vivo, there are limitations that need to be addressed: (1) A possible bias of VF collision pressure since the superior surface stress may yield larger deformations than that of the actual contacting tissue; (2) the use of a non-physiological VF contact geometry (i.e., contact between two spheres); (3) the requirement of calibrated laryngeal HSV to obtain physical dimensions; and (4) the need for VF tissue properties (Young’s modulus and Poisson’s ratio) that are difficult to measure in practice.

The aim of this work is to obtain in vivo estimations of the VF contact pressure directly from laryngeal HSV recordings using a modified version of Hertz contact that addresses the current limitations of the approach. We hypothesize that the apparent penetration and contact surface can be approximated solely from raw HSV videos, which will allow an estimation of the collision forces directly from the observed kinematics. We expect that these direct estimations are simple enough to be applied in a clinical setting. Specifically, we propose a Hertz contact geometry based on two contacting cylinders both to improve the physiological representation of VF contact and to remove the need for a calibrated HSV system. In this formulation, contact pressure is normalized by the tissue properties and a correction factor is introduced to remove the potential bias in estimating contact from employing only a superior view; this enables a focus on same-subject variations.

At the same time, we construct an underlying elastic model with an appropriate stiffness and damping to relate deformation and apparent Hertz tissue penetration and to account for prestress conditions. Finally, to interface the proposed elastic model with a non-colliding condition, a Kalman filter approach is proposed. The approach is contrasted against a separate numerical VF model, silicone VF model experiments, reported experimental data, and in vivo HSV videos to allow for comprehensive insights.

## 2. Contact Pressure Estimation from Laryngeal High-Speed Video

**Figure 1.**
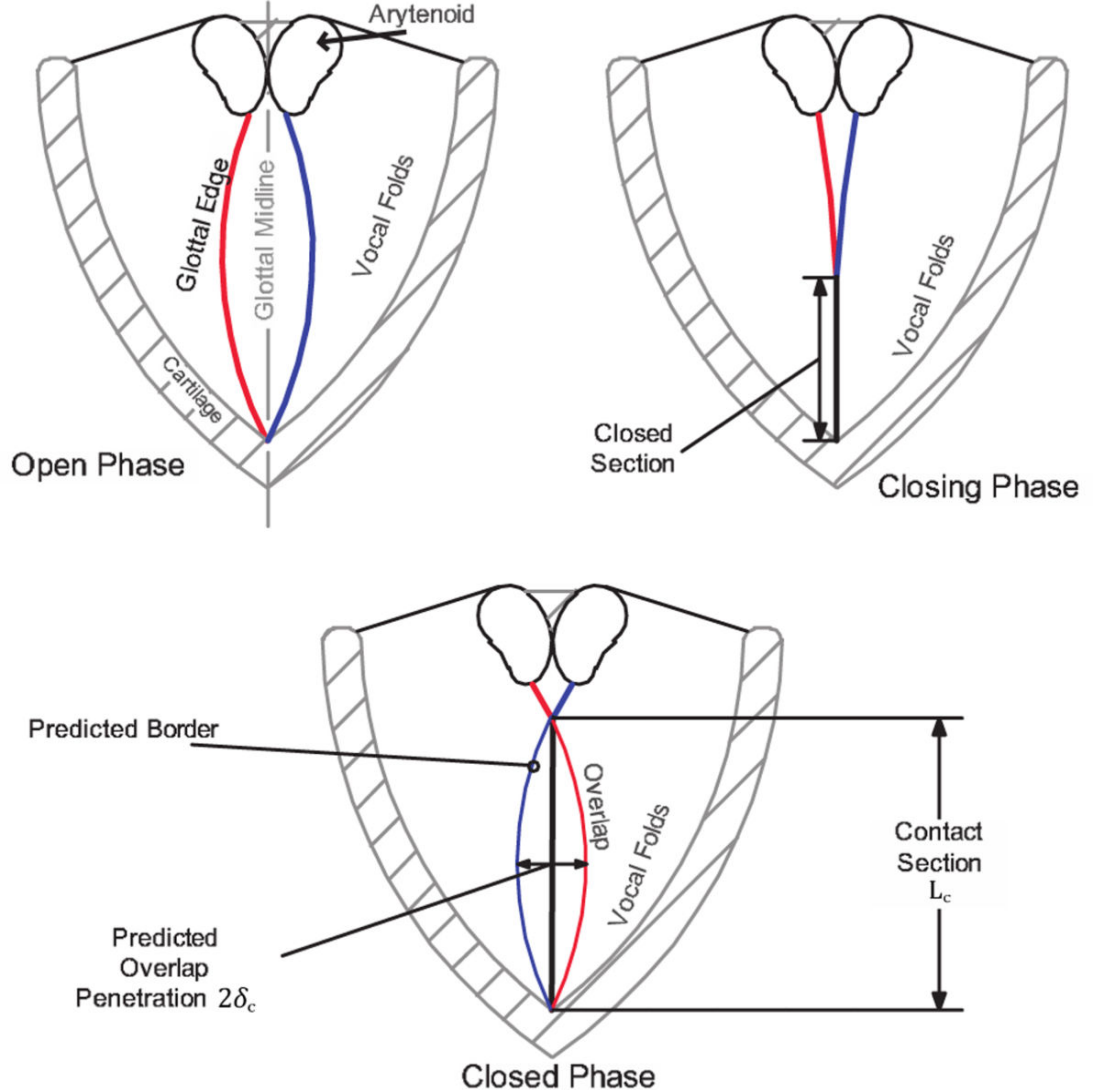
A schematic of a vocal fold (VF) motion sequence in a typical videoendoscopy recording. The colored lines represent the edge estimation of each fold (left in red and right in blue). The fictitious edge overlap during collision shows the estimated penetration depth *δ*_*c*_ and contact length *L*_*c*_.

Herein, we propose a method for estimating the contact pressure experienced by colliding vocal folds based purely on kinematic and geometric information extracted from laryngeal a high-speed video. We refer to this method as Contact Pressure Analysis (CPA). Briefly, the motion of each observed VF edge is extracted from HSV during the open phase of the glottal cycle. The trajectories of the two folds are tracked until collision at which point a simple spring-mass-damper VF model is employed to predict the fictitious overlap of the colliding folds such that the trajectory of each edge matches the observations in the bookending opening phases. It is assumed that the rebounding force due to collision can be estimated using a Hertz contact model, wherein the fictitious overlap is related to the penetration depth for contact between elastic bodies. A schematic of the procedure is illustrated in Figure 1, which shows the VF edges in the open phase and the fictitious penetration depth *5*_*c*_ and contact length *L*_*c*_ during collision.

Implementation of the procedure consists of five stages: (1) *video pre-processing* to remove camera motion effects and to create a uniform viewing orientation; (2) VF *edge detection* to locate the projected medial surface of each vocal fold; (3) VF *curve fitting* to convert the extracted edge location data into a polynomial function; (4) VF *edge tracking* during the open and collision phases; and (5) *contact pressure estimation* through an adapted Hertzian contact model. This section is devoted to expanding on the details of each step in the proposed CPA method.

### 2.1. Video Pre-Processing

Videoendoscopy recordings normally have an undesirable rotation and low frequency movements related to endoscope manipulation. A motion compensation algorithm is first applied to remove low-frequency components [33]. Rotation compensation is achieved by identifying the end-points of the glottis, the line connecting which is used to compute the angle of the anatomical structure with respect to the camera. This measured angle is then used to rotate the image such that the glottis is vertical in the image plane.

A Region of Interest (ROI) centered on the glottis and encompassing the entire glottal length and excursion distance of the VFs is identified for further analysis. A mask is applied to remove information outside of the ROI for each frame in the HSV sequence. A Cartesian coordinate system is defined with *x* oriented horizontally in the image (medial-lateral direction) and *y* oriented vertically (anterior-posterior direction).

The anterior and posterior attachment points of each vocal fold, expressed as (*x*_*a*_, *y*_*a*_) and (*x*_*b*_, *y*_*b*_), respectively, are identified and recorded for later use. The straight line connecting the attachment points for each VF is assumed to be the rest position of that fold.

### 2.2. Edge Detection

Each HSV frame is converted to a grayscale image and a morphological reconstruction operation (imfill in MatLab) is applied to remove bright regions on the VF mucosa. A Prewitt gradient kernel with a user-defined threshold value is employed for edge detection, wherein the phase angle of the gradient vector is used to differentiate between the left and right vocal folds. Specifically, the location of the edge for a given vocal fold at a given *y* location is determined from the weighted average of the magnitude of the gradient function *G* (*x, y*) as

(1) $$(\bar{x},y)=\left(\frac{\sum_{i=1}^{w}x_{i}G(x_{i},\;y)}{\sum_{i=1}^{w}(x_{i},\;y)},y\right),$$

where *w* is the width of the ROI in pixels. We note that data are only considered between (*x*_*a*_, *y*_*b*_) and (*x*_*b*_, *y*_*b*_) for a given fold.

**Figure 2.**
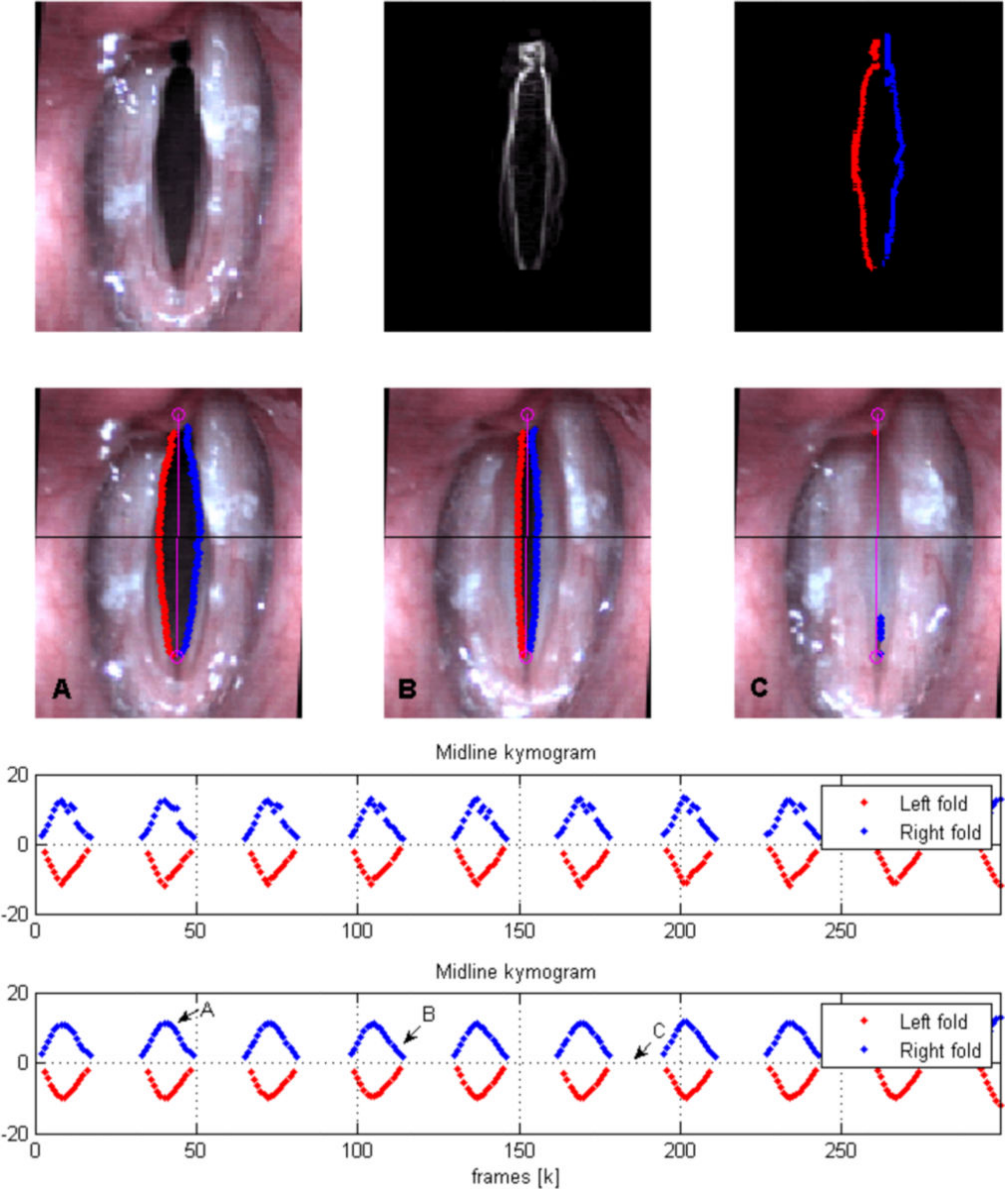
Edge detection example using a HSV recording. Representative steps of the detection process and a mid portion kymography of the glottis are shown. Gradient information is used (first row) to find left and right VF borders. VF edge points are missing during collision (second row). Edge-based kymogram (third row) and its temporally averaged version (fourth row) are also shown. Note that temporal averaging reduces the errors incurred by the edge detection, but it does not complete the trajectory in closing phases.

Lastly, a temporal moving average with a 5 frame uniform kernel is applied to reduce detection errors and noise associated with lighting and/or quantization problems. The first two frames in Figure 2 show the HSV ROI and the output of the edge detection algorithm, respectively.

### 2.3. Curve Fitting

In this step, a polynomial curve of order *p* is fitted to the (*x*, *y*) points from the edge detection, defining the boundary of a given vocal fold with respect to the attachment line (the line defined by the attachment points (*x*_*a*_, *y*_*a*_) and (*x*_*b*_, *y*_*b*_)). A least squares (LS) estimator is used, taking into account the attachment points as root constraints for the solution. Letting *u* and *v* be the coordinates orthogonal to and aligned with the attachment line, respectively, the polynomial model *M* can be defined as:

(2) $$M(v)=\left(\sum_{i=0}^{p-2}\theta_{i}v^{i}\right)\left(v-v_{a}\right)\left(v-v_{b}\right)+u_{a}.$$

where $\Theta ={\left(\begin{array}{llll} \theta_{p-2} & \theta_{p-3} & \ldots & \theta_{0} \end{array}\right)}^{\text{T}}$ is the vector of unknown model parameters with superscript T indicating vector transposition and *u*_*a*_, *v*_*a*_, and *v*_b_ are the attachment point locations in the (*u*, *v*) coordinate system. Note that, assuming a rotation angle *φ* of the attachment line with respect to the vertical axis, the relationship between the (*x*, *y*) and (*u*, *v*) coordinate systems is given by

(3) $$\left(\begin{array}{l} u\\ v \end{array}\right)=\left(\begin{array}{cc} \cos\varphi & -\sin\varphi \\ \sin\varphi & \cos\varphi \end{array}\right)\left(\begin{array}{l} x\\ y \end{array}\right).$$

Using an LS estimator solution, the polynomial parameters contained in Θ are computed as

(4) $$\Theta ={\left(V^{\text{T}}V\right)}^{-1}V^{\text{T}}U$$

where

(5) $$V=\left(\begin{array}{cccc} {\tilde{v}}_{1}v_{1}^{p-2} & \cdots & {\tilde{v}}_{1}v_{1} & {\tilde{v}}_{1}\\ {\tilde{v}}_{2}v_{2}^{p-2} & \cdots & {\tilde{v}}_{2}v_{2} & {\tilde{v}}_{2}\\ \vdots & \ddots & & \vdots \\ {\tilde{v}}_{D}v_{D}^{p-2} & \cdots & {\tilde{v}}_{D}v_{D} & {\tilde{v}}_{D} \end{array}\right);\;U=\left(\begin{array}{c} {\tilde{u}}_{1}\\ {\tilde{u}}_{2}\\ \vdots \\ {\tilde{u}}_{D} \end{array}\right)$$

where ${\tilde{u}}_{l}=u_{l}-u_{a},{\tilde{v}}_{l}=\left(v_{l}-v_{a}\right)\left(v_{l}-v_{b}\right),$ and *l* is an integer ranging from 1 to *D*, with *D* representing the number of points defining the glottal edge detected in the previous stage. The velocity of the glottal edge is embedded in the rate of change of the coefficients Θ, which can be obtained via differentiating between frames as

(6) $${\dot{\Theta }}_{k}=\frac{1}{\Delta t}\left(\Theta_{k}-\Theta_{k-1}\right).$$

where the subscript *k* is the frame index and Δ*t* is the time lapse between successive HSV frames.

The third frame in Figure 2 shows the output of the edge detection scheme. The second row of the figure shows how the edges are tracked from an open phase to a closed phase. Midline kymograms of the VF trajectories during the open phases are plotted at the bottom of the figure for both VFs.

### 2.4. Edge Tracking

The tracking stage treats VF collision as a state estimation problem in the presence of noise and lost data. A Kalman Filter (KF) is implemented to predict the value and rate of change of the coefficients during the contact phase, wherein Θ_*k*_ and ${\dot{\Theta }}_{k}$ are observations for the filter. A dynamical model is needed for the KF process for which we assume a spring-mass-damper representation for each VF connected to their respective attachment lines (defined in the pre-processing stage). The mass in this system is normalized for simplicity. Thus, the structural VF model is given by

(7) $$X_{i,k+1}=AX_{i,k}+D_{k},$$

(8) $$Y_{i,k}=CX_{i,k}+E_{k},$$

$$Y_{i,k}=\left(\begin{array}{c} \theta_{i,k}\\ {\dot{\theta }}_{i,k} \end{array}\right),\;A=\left(\begin{array}{cc} 1 & \Delta t\\ -K\Delta t & 1-b\Delta t \end{array}\right),$$

where *X*_*i,k*_ is the state variable of a particular coefficient *θ*_*i*_ in Θ at the instant *k*, *Y*_*i,k*_ are observations of the state (which we assume is available with *C* as the identity matrix), *D*_*k*_ and *E*_*k*_ are the process and measurement noise (uncorrelated and Gaussian distributed with variances σ_*d*_ and *σ*_*e*_, respectively), *K* is the coefficient stiffness, and *b* is the damping parameter of the model.

The spring-mass-damper model needs to operate at a particular resonance *w*_*r*_, for which we define stiffness *K* and damping *b* values as functions of the control parameters *w*_*r*_ and ξ as

(9) $$K=\frac{w_{r}^{2}}{1-\xi^{2}},\;b=2\xi \sqrt{K}.$$

Typical damping values associated with *ξ* are set between (0 to 0.03). The resonance *w*_r_ is calculated automatically from the kinematic information $\left(\theta_{i,k_{0}},{\dot{\theta }}_{i,k_{0}}\right)$ at the previous instant of impact (*k*_0_), using these as initial conditions. Then, the known analytic solution for the initial conditions is used to coincide the rebounding position $\theta_{i,k_{1}}$ at the end of contact (*k*_1_) with its analytic value. Thus, the proposed scheme adapts the elastic contact properties (stiffness and damping) to match the fundamental frequency of the VFs, which allows for the capture of pre-stress conditions. Thus, we need to find a particular resonance frequency *w*_*r*_ that satisfies

(10) $$w_{r}=\underset{w}{\mathrm{argmin}}\left\|F_{i,k_{1}}(w)-\theta_{i,k_{1}}\right\|,$$

(11) $$F_{i,k_{1}}(w)=\theta_{i,k_{0}}\cos\left(wt_{k_{1}}\right)+\frac{{\dot{\theta }}_{i,k_{0}}}{w}\sin\left(wt_{k_{1}}\right),$$

where $t_{k_{1}}=\left(k_{1}-k_{0}\right)\Delta t.$ The *w*_*r*_ parameter controls the required stiffness of the model to mimic a damped harmonic oscillation during collision.

When the edges are visible, this contact model is not needed and, thus, is no longer used. To define when KF estimations are required, we calculate a “non-detected points ratio” *λ* and an uncertainty factor *ρ* as

(12) $$\lambda_{k}=\frac{D_{T}-D_{k}}{D_{T}},\;\rho_{k}=\frac{1}{1+e^{-\beta \left(\lambda_{k}-\gamma \right)}},$$

where *D*_*T*_ is the upper bound of all possible detected points at the edge, *D*_*k*_ is the actual detected points, *β* is a gain factor, and *γ* is an uncertainty threshold.

When *D*_*k*_ is low, for example, *λ*_*k*_ tends to be larger than the uncertainly threshold and *ρ*_*k*_ goes to one; that is, there are too many lost data points, the polynomial fit is poor, and thus, KF estimations are needed. The KF implementation considers the uncertainty factor *ρ*_*k*_ from Equation (12) and follows

(13) $${\hat{X}}_{k+1|k}=A{\hat{X}}_{k|k},$$

(14) $${\hat{X}}_{k|k}=\left(I-J_{k}C\right){\hat{X}}_{k|k-1}+J_{k}{\bar{Y}}_{k},$$

(15) $$J_{k}=P_{k|k-1}C^{T}{\left[CP_{k|k-1}C^{T}+P_{E}\right]}^{-1},$$

(16) $$P_{k+1|k}=AP_{k|k}A^{T}+P_{V},$$

(17) $$P_{k|k}=P_{k|k-1}-\left(1-\rho_{k}\right)J_{k}CP_{k|k-1},$$

(18) $${\hat{Y}}_{k}=C{\hat{X}}_{k|k-1},$$

where the coefficient index *i* has been dropped for simplicity. The estimated outputs are defined as ${\bar{Y}}_{k},$ which are a linear combination of the observations $Y_{k}$, and KF estimated observations ${\hat{Y}}_{k}$ that are related through

(19) $${\bar{Y}}_{k}=\left(1-\rho_{k}\right)Y_{k}+\rho_{k}{\hat{Y}}_{k}.$$

We note that *ρ*_k_ controls the behavior of the filter; by selecting the best available curve coefficients, the evolution of each curve is completed smoothly throughout collision.

**Figure 3.**
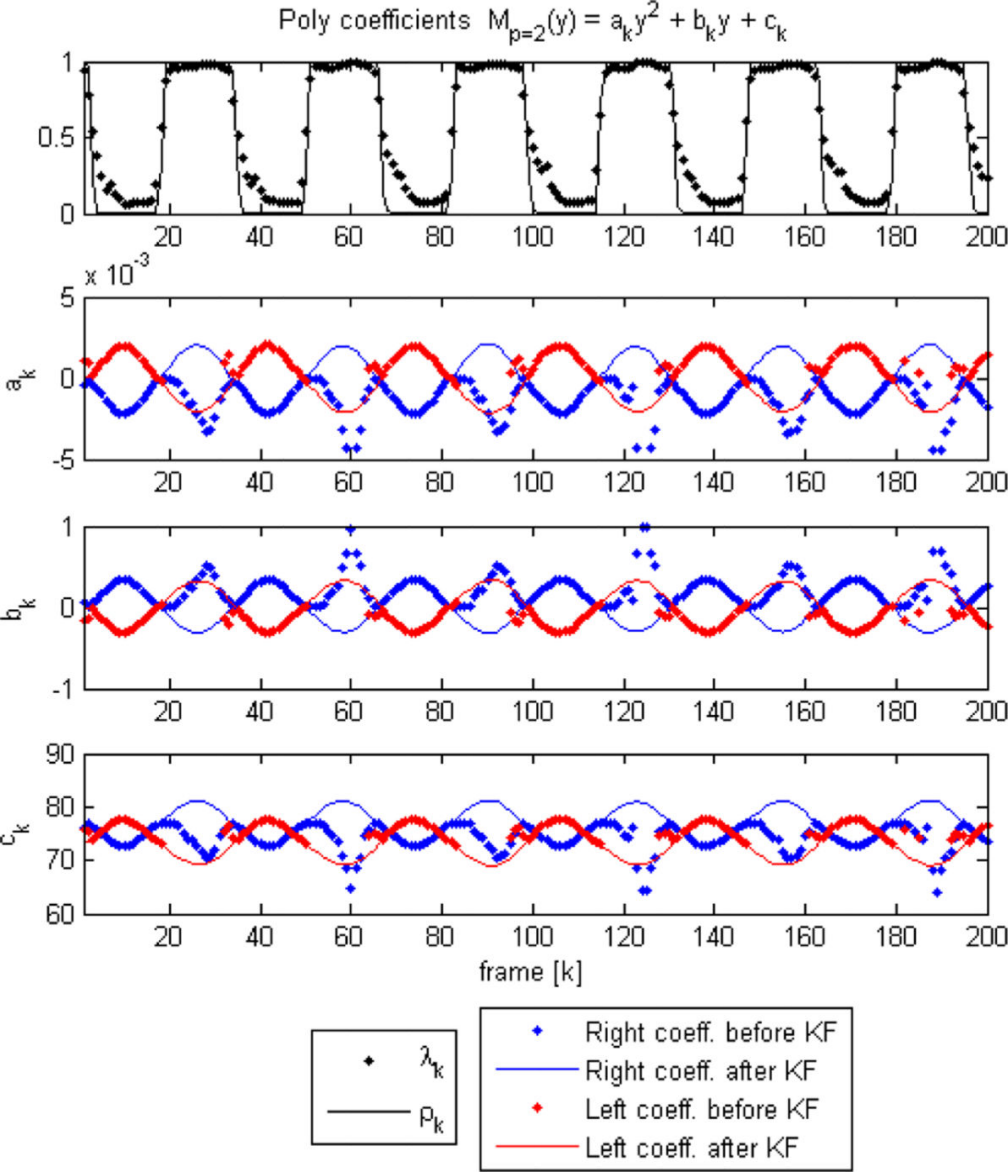
Coefficient tracking using the Kalman Filter(KF) during closure. Quadratic curves are employed (*p* = 2). The estimates of Θ_*k*_ are converted to standard quadratic coefficients as ${\left(a,b,c\right)}_{k}={\left(1,-\left(y_{a}+yb\right),x_{a}+y_{a}y_{b}\right)}_{k}\theta_{0,k}$ and are plotted across time. The polynomial coefficients of each fold are shown before and after the KF edge tracking module, with the ratio *λ*_*k*_ and uncertainty factor *ρ*_*k*_ (only the uncertainty of the right side is shown for simplicity). The VF description fails when collision occurs, making registered coefficients not valid at certain times. The KF completes the temporal sequence of the fitted model by making predictions of its location during the collision phases (high *ρ*_*k*_ values).

**Figure 4.**
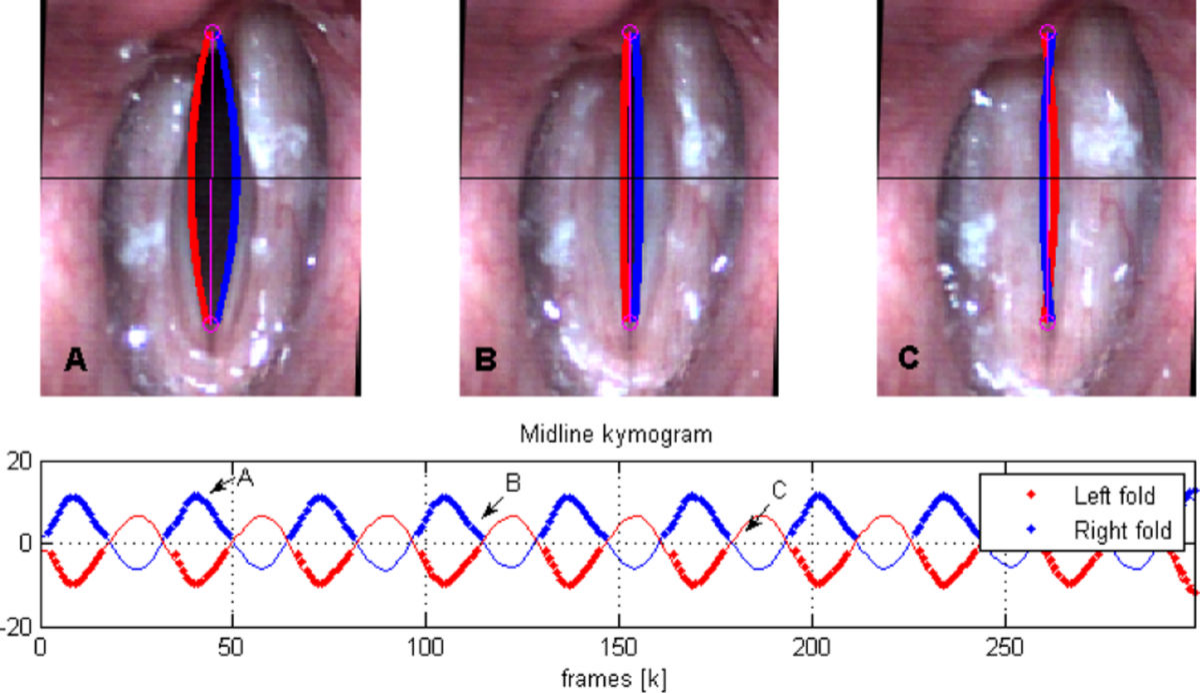
KF Edge tracking result. Three representative instants of the closure and a midline kymogram are shown. An overlap between VF is now visible during contact phases despite the lost of detected points in the detection stage.

An example of polynomial fitting during open phases and KF during closure for vertically oriented attachment lines (that is, *φ* = 0) with *p* = 2 is shown in Figure 3. In this case Θ_*k*_ = *θ*_0,*k*_ and the plotted coefficients (*a, b, c*)_*k*_ are computed as ${\left(1,-\left(y_{a}+y_{b}\right),x_{a}+y_{a}y_{b}\right)}_{k}\theta_{0,k}$. The top row shows a plot of *λ* and *ρ*, indicating when edge tracking is used (high *λ*) versus when it should fail (low *λ*) and thus requiring KF to complete the cycle. We note that the coefficients (*a, b, c*) are prone to producing poor fits during collision, since the LS estimation is not well-conditioned when *D* decreases suddenly due to failure of the edge detection algorithm during closure. As such, during collision, we lose the VF location. As shown in Figure 4, the KF handles the loss of detection points by switching to model predictions considering the last position and velocity. When collision ends, the uncertainty factor drops and the estimation returns to the detected edges from the previous stage.

### 2.5. Contact Pressure Estimation

Hertzian contact theory [26,27] describes the stresses occurring in elastic bodies in contact when the local deformation is small in comparison to the characteristic scale of the body. As such, the contact mechanics can be described in terms of the effective contact surface and apparent penetration, that is, the degree to which a body with the same geometry would “penetrate” the contacting body under the same displacement condition [25]. As a first-order approximation, we herein assume that the contact area between opposing vocal folds begin as a line in the inferior-superior direction, with the contact area increasing as the folds progress through the collision event (refer back to Figure 1 for the collision geometry). Noting that the vertex of a quadratic (*p* = 2 from the previous sections) is locally circular, we model the collision event as a Hertzian contact between two cylinders with parallel axes, assuming linear elasticity.

Following Boresi et al. [34], the maximum contact pressure *P*_*c*_ experienced by colliding cylinders is given by

(20) $$\frac{P_{c}}{E^{*}}=4\alpha \frac{\delta_{c}}{L_{c}},$$

where *δ*_*c*_ is the penetration depth and *L*_*c*_ is the contact line length observed from the HSV; refer to Figure 1 for a schematic. The parameter *α* is a correction factor to account for VF inferior-superior contact bias; we note that this is not included in standard derivations of Equation (20). This factor is initially assumed to be unity, meaning that there is no bias due to the contact geometry assumption; however, it will be later calibrated as part of the numerical validation.

The parameter E* in Equation (20) is the effective Young’s modulus, defined as

(21) $$E^{*}=\frac{1}{\frac{\left(1-v_{1}^{2}\right)}{E_{1}}+\frac{\left(1-v_{2}^{2}\right)}{E_{2}}}=\frac{E}{2\left(1-v^{2}\right)},$$

where *E* is Young’s modulus, *v* is Poisson’s ration, and the subscripts 1 and 2 indicate the two VFs, thus allowing consideration of cases wherein the two VFs have different material properties. For the remainder of this study, however, the two VFs are assumed symmetric with regards to tissue properties, that is, *E*_1_ = *E*_2_ = *E* and *V*_*1*_ = *V*_2_ = *v*, and E* is given by the simplified right hand side of the equation. We note that the normalized maximum contact pressure is purely a function of measured quantities. Alternative geometric models for the vocal folds, such as spherical contacting bodies, result in similar expressions for *P*_*c*_/E^*^, though they generally include additional geometric factors, such as the thickness of the VFs, which are not readily measured with HSV. It is also important to note that the result is independent of the cylinder radius in contrast to other shapes (e.g., colliding spheres).

Equation (20) yields the dimensionless maximum contact pressure. Determination of a dimensional value requires knowledge of the elasticity of the subject’s vocal folds, which is not directly assessable from HSV alone. However, the aim of this project is to develop a tool that allows for simple contact estimations based solely on kinematic data. If quantitative values for the contact pressure are desired, either additional analysis methods must be employed, such as inverse analysis techniques [35,36], or population-based values from the literature may be employed. The dimensionless maximum pressure is of a direct value for comparative evaluations performed on a single subject or, using population-based values, as a first cut approximation of the collision forces.

The final stage of CPA consists of extracting the apparent penetration *δ*_*c,k*_ from the curve representation for each HSV frame. The predicted coefficients Θ_*k*_ from Equation (19) are used to calculate the polynomial parameters for each VF. Finally, we take these polynomial curves to compute the next sequence

(22) $$\Delta {\hat{x}}_{j,k}=M\left(y_{j,k};\Theta_{k}^{\text{left }}\right)-M\left(y_{j,k};\Theta_{k}^{\text{right }}\right),$$

(23) $$\delta_{k}=\max\left\{\Delta {\hat{x}}_{j,k},\forall j|\Delta {\hat{x}}_{j,k}>0\right\}.$$

## 3. Methods

In this section the CPA method is validated against pressure estimations from other methods. Specifically, we validate quantitatively against self-sustained VF model simulations and silicone models and semi-quantitatively against experimental data from in vivo recordings with laryngeal HSV of a single subject under various conditions.

### 3.1. Numerical Validation

Synthetic videos were obtained from a numerical model of voice production referred to as the *Modified Body Cover Model* (MBCM), which is a modification of existing VF models [5,37] to better serve this study. The model features posterior and membranous glottal openings (PGO and MGO) implementations and a triangular (non-plane) intraglottal mass surface representation, which allows for modeling the variations of the contact area and pressure during closure phases in the anterior-posterior direction. The model is fixed on the anterior and posterior VF locations, and the medial portions are considered point masses. The shape of each fold is defined by a linear interpolation between the anterior, medial, and posterior points. When the cover masses are in contact, the resulting overlapping shape has a distributed contact load that is used to calculate the lumped contact pressure interaction between the cover masses. In particular, the contact depth, contact area, and contact pressure values are extracted from the lower cover masses to conform to the benchmark data set. Glottal opening areas are controlled by parameters governing the thyroarytenoid configuration, with the possibility to define incomplete glottal closure scenarios with PGO and MGO gaps. During collision, the MBCM masses overlap as in traditional lumped element VF models [38]; the penetration depth of the MBCM is observable and serves as a target for analysis of the proposed CPA method.

The MBCM model was used to simulate two sets of test cases, one with complete glottal closure phonation (TEST1) and the other with incomplete glottal closure phonation (TEST2). Each test has the same muscle activation, posturing, and vocal tract configurations but differ in the PGO/MGO gaps, which are zero in TEST1 and non-zero in TEST2. For each set, 53 videos with different subglottal pressure values, ranging from 700 to 2000 Pa were obtained. In both test sets, muscular activity was fixed to *a*_*ta*_ = 0.5 (thyroarytenoid), *a*_*ct*_ *=* 0.1 (cricothyroid), and *a*_*lc*_ = 0.495 (lateral cricoarytenoid) [39]. Grayscale frame sequences with a transverse view of the MBCM model showing a glottal gap representation were generated. Point locations of each mass were interpolated with parabolic curves to reproduce the glottal gap. Each video frame is 300 × 300 pixels and the frame rate is 8750 fps.

The CPA algorithm is computed on each video with the same configuration setup. The attachment points for these tests are defined by the anterior and posterior rest positions of the MBCM’s low masses, and the video orientation is fixed; as such, the preprocessing stage is not needed. Validation with the synthetic videos consists of comparisons of penetration and collision pressure between the MBCM model and the CPA predictions.

### 3.2. Silicone Model Experiments for Validation

**Figure 5.**
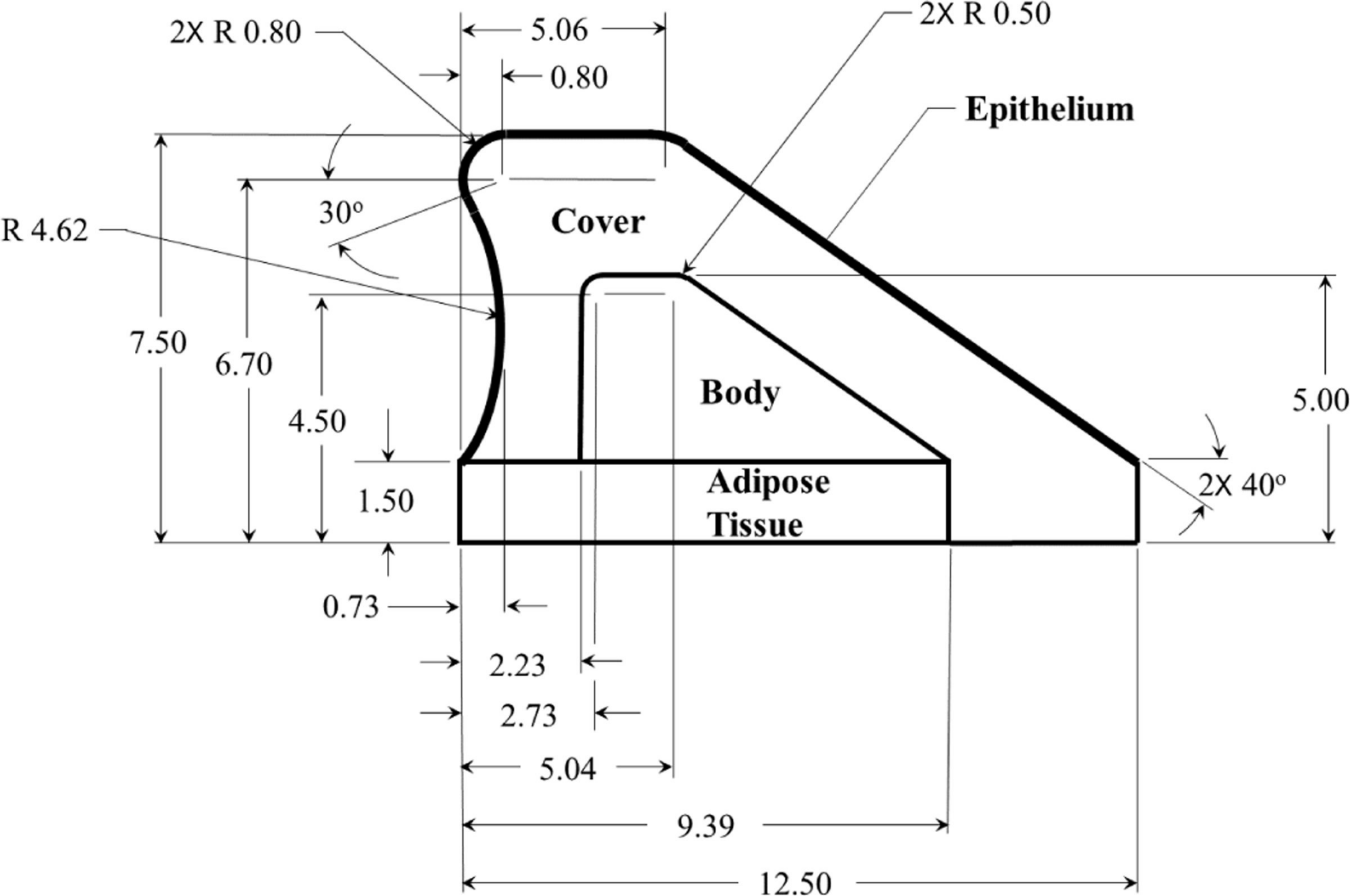
Geometry and dimensions of the synthetic vocal fold model. All reported dimensions have units of cm.

**Figure 6.**
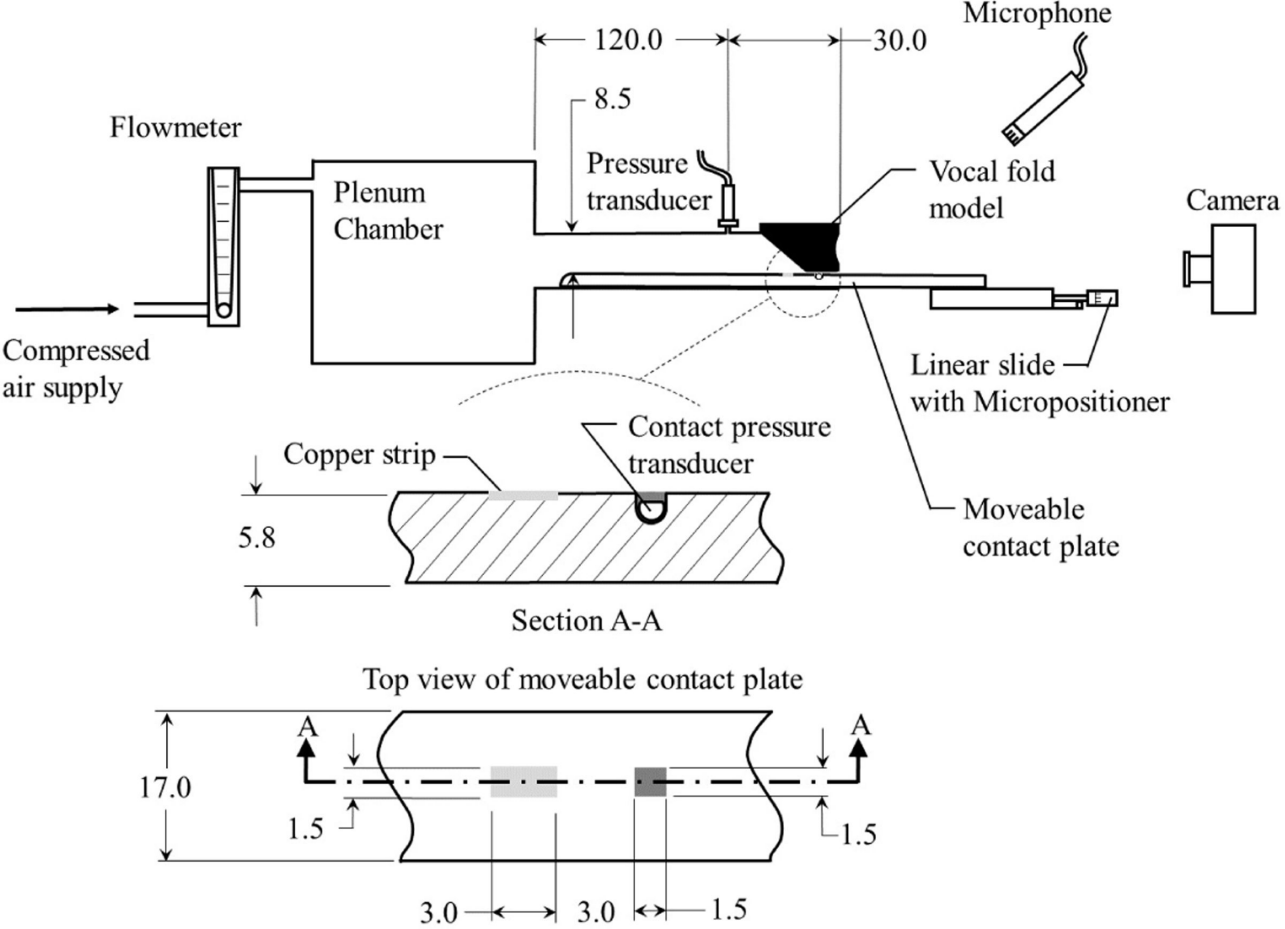
A schematic of the experimental flow facility. All dimensions shown are in mm.

Contact dynamics in silicone, self-oscillating vocal fold models are used for validation of the Hertzian contact approach. The silicone models utilize the same material and follow previously developed approaches for a multi-layer fabrication of silicone vocal fold surrogates [40–43]. Details of the geometry utilized for the models are shown in Figure 5, and the corresponding modulus of elasticity for each layer are presented in Table 1. The models are inserted into a hemi-laryngeal flow facility, which is driven by regulated, compressed air. Details of the flow facility are presented in Figure 6. Airflow passes through an in-line Dwyer RMC 103-SSV flow meter before entering the flow facility. The flow facility consists of a lung plenum with a 0.03 m^3^ volume, that has acoustically treated interior walls to minimize acoustic reflections. The plenum exits into a 2.50 cm wide by a 0.75 cm high subglottal channel. The synthetic vocal folds are mounted at the exit of the subglottal tract. A static pressure tap measuring 1.25 mm in diameter is placed upstream of the exit and is connected to a Kulite ET-3DC pressure transducer that monitors the unsteady subglottal pressure. Details of the vocal fold geometry and the stiffness of each corresponding layer are detailed in Figure 5.

During oscillation, the medial vocal fold surface contacts the lower wall of the subgtlottal tract, which has a movable contact plate insert to enable a direct measurement of the contact pressure and location. The contact pressure is measured by a Millar Mikro–Cath pressure transducer that is flush mounted in the wall of the contact plate and is embedded in silicone such that the upper surface of the tongue remains flat. The pressure transducer is recalibrated while embedded in the silicone via static weight tests that measure the contact force and area of silicone discs resting on the surface of the tongue. In addition, the frequency response of the embedded transducer was measured to be greater than 3.8 kHz.

To measure the precise location of contact, the procedure first proposed by Reference [44] is followed. A 1.50 mm wide (anterior-posterior direction) by 3.00 mm long (inferior-superior direction) copper strip is mounted flush to the surface of the tongue and is positioned precisely 3.00 mm inferior to the pressure transducer. The vocal folds models are covered in graphite powder and, together with the copper strip, are wired into one leg of a Wheatsone bridge. Each of the other three legs of the Wheatstone bridge are comprised of a 330 kΩ resistor. In this manner, when the vocal folds contact the copper strip, the resistance through the VF leg of the circuit, which is determined by monitoring the output voltage from the circuit via LabView^®^ PICe-6321 data acquisition card, decreases, and contact is identified. The movable contact plate extends beyond the exit plane of the vocal folds and is attached to a micropositioner (Thor Labs PT1) so that it can be precisely positioned in the inferior-superior position to determine the contact location. Once the precise position of contact is identified, the plate is adjusted inferiorally/superiorally by 3.0 mm, which corresponds to the offset distance between the copper contact strip and the pressure transducer, thereby ensuring that the contact pressure sensor is centered within the region of contact, where the contact measurements are performed. Contact pressure measurements are acquired at a sampling frequency of 80.0 kHz for 0.75 s and are then sorted and averaged based on the period of oscillation. The peak contact pressure is then identified as an average across all of the acquired cycles.

A high-speed video from a superior view of the vocal folds is simultaneously acquired and synchronized with the pressure acquisitions at 20,000 fps. Data are acquired for three separate moduli of elasticity for the model vocal fold cover: 0.95 kPa, 1.65 kPa, and 2.45 kPa. At the lowest cover value, HSV is captured at a subglottal pressure of 2.15 kPa. For a cover module of elasticity of 1.65 kPa, HSV is captured at subgltottal pressures of 2.20–3.40 kPa in increments of 0.20 kPa. Finally, at the highest value, HSV is acquired for a subglottal pressure of 3.40 kPa.

### 3.3. In Vivo Experiment

The HSV recordings are used as a proof of concept for in vivo clinical scenarios. Three phonation gestures with increasing loudness (soft, normal, and loud) in a normal subject were recorded. Each HSV has about 2670 frames of 300 × 300 pixels at 8000 fps, recorded with a high-speed camera (SA-X2, Photron, Tokyo, Japan) connected to a rigid endoscope (9106, KayPentax, Montvale, NJ) using a 35-mm C-mount adapter and a xenon light source (7152B, KayPentax, Montvale, NJ). Each loudness gesture was accompanied with its corresponding *Sound Pressure Level* (SPL), obtained with a condenser microphone (ECM8000, Behringer, Willich, Germany) positioned 8 cm away from the mouth and calibrated with a Sound Level Meter (NL-42, Rion, Tokyo, Japan). The procedure was performed under informed consent, following the IRB-approved protocol for the FONDECYT project 1151077. The experiment with these in vivo HSV consists of semi-quantitative comparisons between the contact pressure reached by the patient, taking normal loudness as the reference point. HSV dimensions and CPA configuration parameters are not calibrated; as such, the same CPA values from the synthetic videos in the prior section were used for this experiment. The latter is particularly useful to contrast the proposed Hertz contact output in physical units (e.g., Pa) against the reported data [16,20–22].

All CPA configuration parameters are shown in Table 2 for both synthetic and in vivo HSV experiments. Note that the material properties are not needed to estimate the normalized pressure but are listed here to allow for a direct comparison with prior studies in the experimental validation.

## 4. Results

### 4.1. Numerical Validation

**Figure 7.**
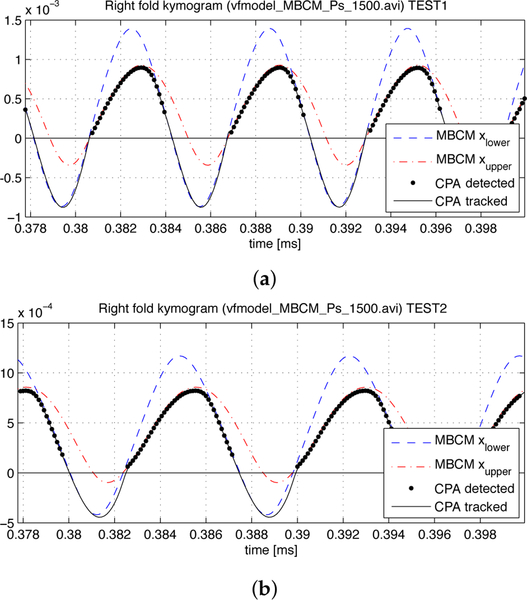
Right side kymograms with upper and lower mass displacements from Modified Body Cover Model (MBCM) and CPA trajectory predictions. Two particular simulations from (**a**) TEST1 and (**b**) TEST2 at *Ps* = 1500 Pa are shown. CPA-detected and -tracked trajectories were obtained only by processing the videos. CPA-detected points were used to reproduce the lower mass penetration in both cases, considering their kinematics.

Examples of predicted VF trajectories for both synthetic tests are shown in Figure 7. The CPA method detects the minimum glottal distance and is capable of reproducing the apparent penetration between the tissues with reasonable accuracy. We note that, due to the phase lag between the upper and lower masses, inferior mass impacts are tracked. The MBCM shows a relatively low penetration of the upper VF mass, and thus, it contributes less to the total contact force in these simulations. CPA is found to be sensitive to different penetration depth values, predicting correctly more overlap displacement in complete closure phonation (TEST1) than incomplete closure phonation (TEST2) at equal subglottal pressures.

**Figure 8.**
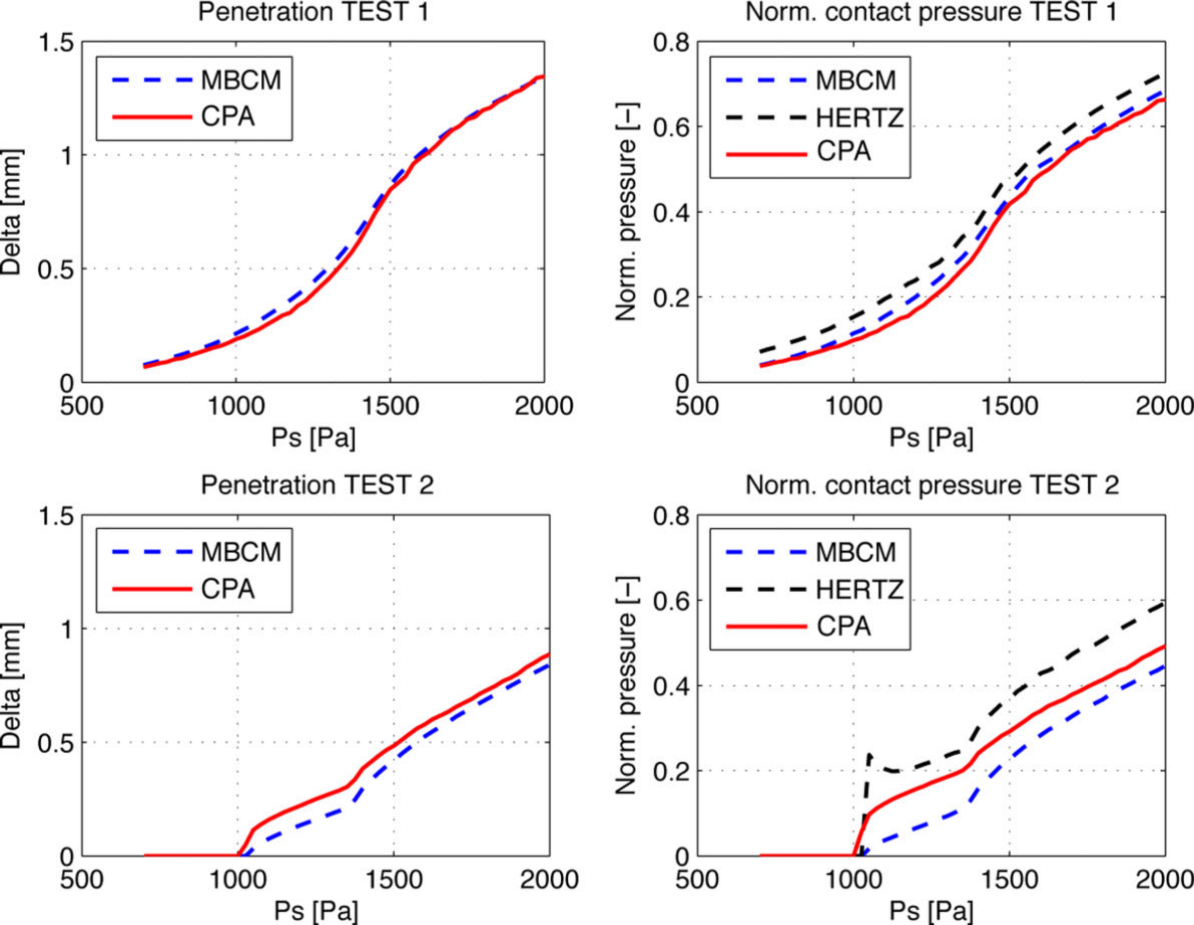
Numerical validations for both complete (top row; TEST1) and incomplete (bottom row; TEST2) glottal closure scenarios. Apparent penetration (left column) and normalized pressure (right column) are presented for the reference model MBCM, the idealized HERTZ contact accounting for the superior and inferior tissue, and the proposed CPA scheme using the superior view only. Note that subglottal pressures below 1000 Pa were not sufficient to cause VF contact under incomplete glottal closure scenarios.

Results from synthetic tests are shown in Figure 8. The apparent penetration and normalized pressure are compared with simulated data. Contact pressure was calculated by taking the average of all peak values. Though not shown for brevity, the effective contact area in both tests was overestimated, as expected, since the CPA method considers an area with full contact along the VF thickness (i.e., there is no inferior-superior variation in contact).

Included in the contact pressures plotted in Figure 8 is a line labeled “HERTZ”. This line was obtained by using the actual inferior and superior mass overlap and contact area from the MBCM model, rather than that estimated through the CPA, in Equation (20). Due to the consistent positive bias observed in the idealized HERTZ pressure values, it is reasonable to conjecture that bias errors arise from a missing term or formulation in the Hertz equations for obliquely contacting bodies. Thus, we calibrated the correction factor *α* in Equation (20) to compensate for this effect through an optimization scheme for all simulations, yielding *α* = 1.679. This factor is used in all subsequent results and discussions to compensate for inferior-superior variations in contact area geometry.

The results from TEST1 shown in Figure 8 suggest that the corrected Hertzian model is an accurate contact estimator for a complete closure phonation scenario. Here, penetration and contact pressure predictions have similar values to the MBCM. MBCM pressure is well-predicted by HERTZ, and the CPA estimation errors for this test remain bounded when compared with the numerical simulations. The ideal prediction by HERTZ of TEST2 in Figure 8 shows some error compared to the MBCM results, which suggest that the Hertz model is less accurate in estimating the contact pressure for incomplete glottal closure configurations.

### 4.2. Silicone Model Validation

The silicone model experiments allow for a separate validation using a completely different model conception, which is desirable to eliminate any possible bias from our prior numerical validation. However, a separate challenge occurs in this scenario. State-of-the-art silicone VF model designs are multi-layered, with material properties that closely resemble human tissue properties. Thus, the estimation of the “equivalent” Young modulus for the purpose of the Hertz contact model is not straightforward. Studies in silicone VF models and human larynges have demonstrated that the estimation of the Young modulus is a function of the indentation method [45] and that it is likely to be affected by boundary conditions. Furthermore, the colliding portion of the VFs is a function of the vibrating tissue, which can involve different layers under different conditions, thus changing the equivalent elastic modulus involved during contact. To address this issue, we propose estimating the equivalent elastic modulus of the silicone model using the dynamic structure of the proposed Hertz and available measured pressure data and leaving some cases out for cross-validation. In particular, the recordings obtained with the modulus of elasticity with more data points, namely *E*_*cover*_ = 1.65 kPa, are used to estimate the equivalent Young modulus, as shown in Table 3. For this purpose, the peak contact pressure measured from the intraglottal Millar pressure probe is contrasted with the dimensionless term from Equation (20) to estimate the equivalent Young modulus. Note that we also use the correction factor *a* = 1.679 obtained in the prior numerical validation.

The resulting modulus of elasticity from Table 3 has a mean value of 15.1 Pa and a standard deviation of 7.2 Pa. The relatively large variability in the estimated values is due to various factors, including the hemilarynx configuration, errant shadowing, bright light reflections, etc. These effects were more or less critical in some cases, up to the point where it was not possible to accurately analyze the video (e.g., for the case of subglottal pressure equal to 3 kPa in Table 3). Efforts will be devoted to minimize these imaging problems in subsequent studies.

As a first-order approximation, we assume that there is a linear relation between the equivalent elasticity and the cover elasticity, and if we normalize the former with the latter, we obtain a dimensionless elastic term that can be used to predict the equivalent elastic moduli for other conditions. Using this idea, we estimate that the silicone model with a cover stiffness of 0.95 kPa has an equivalent elasticity of 8.7 ± 4.1 kPa (mean and standard deviation) and that the silicone model with a cover stiffness of 2.45 kPa has an equivalent elasticity of 22.4 ± 10.7 kPa (mean and standard deviation). We then use these values to contrast the predicted and measured contact pressures, as shown in Table 4. It is interesting to note that there is almost a factor of 10 between the cover stiffness and the equivalent elastic module needed for Equation (20).

Table 4 shows that there is an excellent agreement between the mean peak contact pressure predicted with the proposed approach and the one measured using an intraglottal pressure probe. However, there is a considerable variability in the estimates due to aforementioned image processing difficulties in our silicone experiments. Thus, further experiments with more controlled lighting conditions and more model configurations (elastic moduli and subglottal pressures) are suggested for future studies.

### 4.3. In Vivo Example and Contrast with Prior Studies

**Figure 9.**
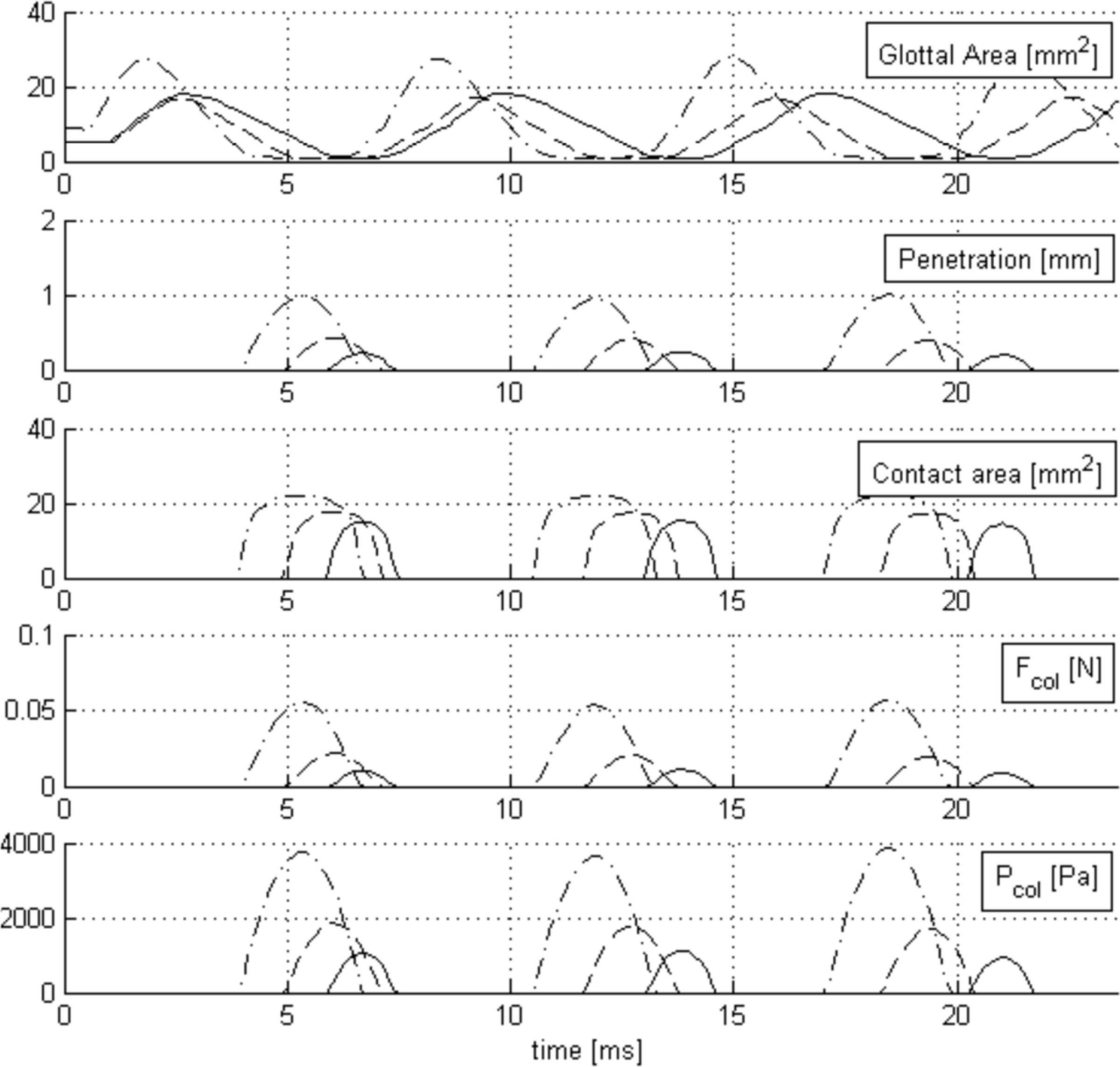
Hertzian pressure estimations for in vivo HSV recordings. Glottal area, penetration, and contact area estimates for soft (solid line), normal (dash line), and loud (dash-dot line) gestures are shown. Increasing impact slopes of glottal area are observed with increasing loudness. Increasing penetration depth and estimated normalized contact pressure are observed with louder gestures.

The glottal area from HSV at different loudness levels is presented in Figure 9 along with predictions of the penetration depth and normalized contact pressure from the CPA procedure. We utilize the material properties listed in Table 2 to obtain contact pressure in physical units. Note that the *E* = 24.75 kPa, which implies that E* *=* 16.5 kPa, and if we use the same correction factor as in the silicone models, we would have $E_{cover}^{*}=1.80\;\text{kPa},$ which is in the range of experimental data [45]. From the glottal area waveform, it is evident that the phonation frequency increases with increasing loudness and that the peak glottal area and rate of closure is highest for the loud gesture. The normal and soft gestures exhibit approximately the same peak glottal area and similar closure rates, thus offering a diverse set of cases to study the robustness of CPA. The penetration and normalized contact pressure estimations exhibit increasing peak values with increasing loudness, as intuitively expected.

Phase differences in the VF positions during contact were observed, exhibiting more displacement and delay in the right VF of the subject. The fictitious overlap observed is not symmetric and depends on the kinematic behavior of each VF border. CPA was sensitive to different VF displacement magnitudes, depending on contact time and impact velocity, which is noted in the glottal area amplitude and rate of change. Abrupt closure develops more deflection and, therefore, presents higher penetration values.

CPA estimations are further quantified in Table 5. The mean peak values of contact pressure for absolute and relative values to the normal gesture (in brackets) are shown. Comparing the contact pressure with the normal loudness scenario, the louder case is at least two times higher and the softer case is roughly a half of it. This implies that doubling the contact pressure results in roughly +10 dB in the SPL in this scenario. Some previous evidence of this variations was documented by Gunter et al. [8] and Verdolini et al. [7], where comparable values were reported across similar loudness intensities.

**Figure 10.**
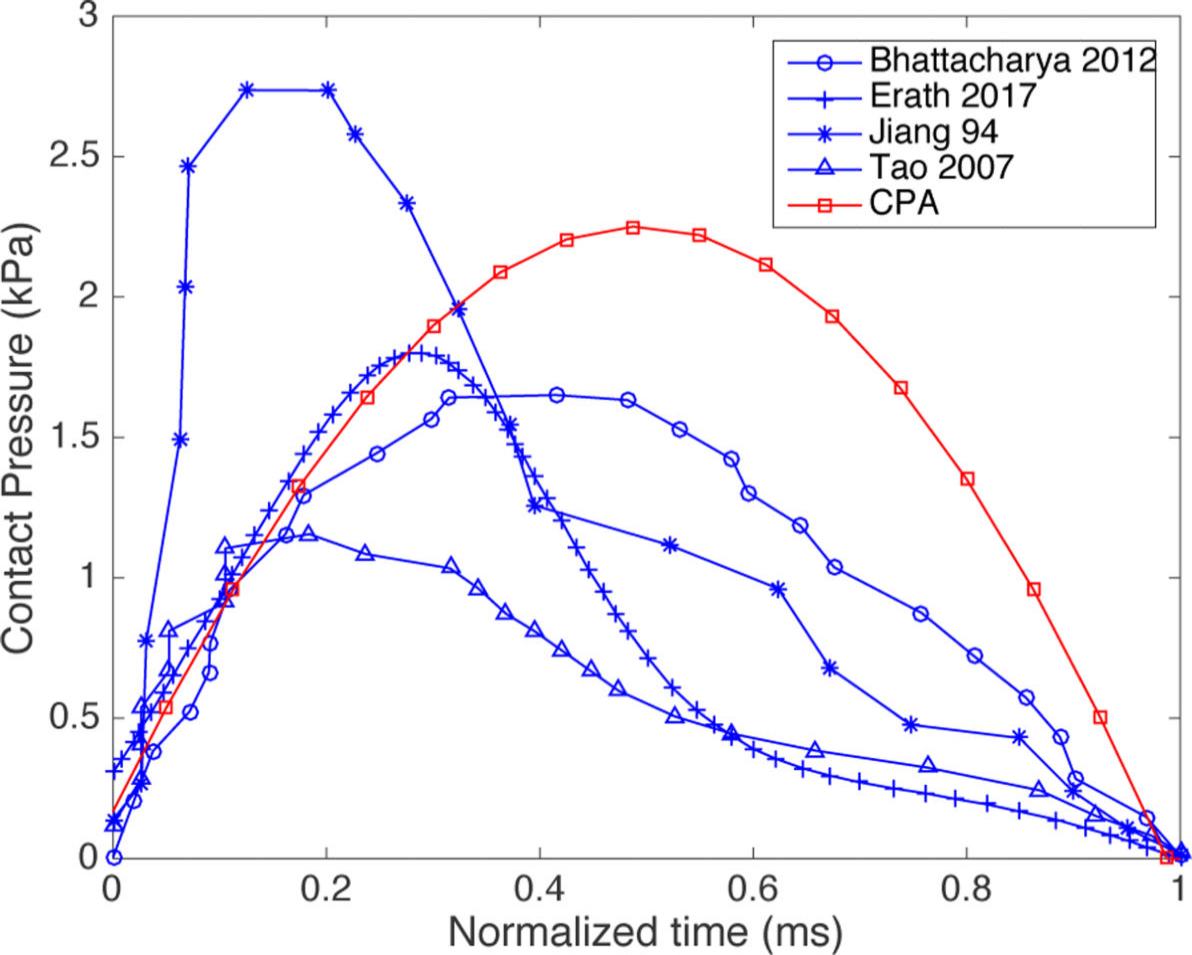
Validation of the proposed Hertz contact model (red lines) against published data (blue lines). For ease of comparison, the time axis was normalized to maintain a contact duration of 1 ms.

A further quantitative comparison is obtained by contrasting CPA-predicted contact pressure estimations with the published data of time series of contact pressure from hemilarynx experiments [16], finite element models of vocal fold collision [20,21], and an analytical viscous contact model [22]. As an example, we use the normal loudness condition to better resemble these prior studies. Figure 10 shows the contrast of the proposed Hertz contact model (red line) against prior studies (blue lines) [16,20–22]. Given that the duration of the contact pulse is a function of the fundamental frequency, contact pulses can vary between 0.8 and 1.8 ms in these cases. For ease of comparison, the time axis was normalized in Figure 10 to maintain a contact duration of 1 ms. It can be seen that the resulting pulse yields comparable magnitude, although it does not capture the waveform asymmetry that some of prior studies report. This limitation, however, does not hamper the correct estimation of the maximum peak in the contact pressure, which is considered more clinically relevant. In addition, note that this pulse asymmetry is not observed in one of the prior studies using finite element models [21]. Note that the resulting contact pressure waveform is symmetric due to the underlying lumped mass model in the Kalman filter, which uses a linear spring as a first approximation to describe the overlap penetration. Nonlinear spring models could be explored in future studies to capture the role of hysteresis in the contact pressure. Based on these results, we conclude that the proposed method constitutes a simple yet valid method for a first-order approximation of the in vivo contact pressure estimation.

## 5. Discussion

The fundamental tenet of CPA is that sufficient information regarding the collision mechanics is embedded in the vocal fold kinematics, which is accessible through high-speed videos to obtain reasonable first-order estimates of contact pressure. That is, CPA is founded on the principle that impact velocity and contact time are sufficient to assess collision pressure magnitudes. Our validation using silicone vocal fold models illustrated that there is an excellent agreement between the mean predicted contact pressure, although with some nonnegligible variability due to image processing difficulties. Agreements with prior numerical studies [20–22,46,47], experimental measurements in humans [6–8], and excised hemilarynx studies [16] also have been found. This supports that the Hertz contact framework is appropriate for the goal of estimating contact pressure directly from the kinematic information, as described in the CPA framework.

There are clinical arguments that suggest that aerodynamic parameters, such as *Maximum Flow Declination Rate* (MFDR), are related to collision pressure and stress [1,5]. Similar relative changes in aerodynamic-based assessment parameters with loudness for normal subjects have been observed in prior studies [47] as found with the contact pressure estimates in this study. Numerical models support this relation between MFDR and collision pressure [5,37], but the conjecture is still not proven and only theoretical studies that evaluate this are known. That said, our case study supports the idea that CPA is capable of discriminating the increasing effect of collision pressure with loudness in actual in vivo scenarios despite the lack of aerodynamic or other additional information in the recordings.

The proposed Hertz contact model, which, by virtue of employing cylinders, implicitly assumes uniform and simultaneous contact along the thickness of the VFs, has the advantage of not requiring additional subject-specific information in order to estimate the normalized contact pressure. Geometric variations in the inferior-superior direction, however, do influence the contact pressure, as evidenced by the estimated value of *α* in Equation (20) differing from one. The value employed in this study was obtained from the numerical validation phase through a comparison with the reduced order model performance. It remains unclear how universal this geometric correction factor may be and requires additional data sets. It is unlikely that the value of *α,* even if it is not relatively constant between individuals, will change dramatically, however. Similarly, to obtain absolute measures of contact pressure from CPA, one requires additional patient-specific information, namely tissue elasticity. This value can change significantly between subjects or even between vocal gestures. However, even though there could be a bias in the exact magnitude of the collision pressure due to either *α* or elastic modulus, the relative changes within the same subject are expected to be well-represented. Having access to these simple estimates can be of great interest for clinical practices to easily assess the effectd of therapy or surgical procedures on a given subject, where the unknown tissue and/or geometric parameters remain the same.

There are several parameters that are user-specified when employing CPA: the threshold value for the edge detection of the VFs; identification of the attachment point locations, which dictates the “rest” positions of the folds in model space; and the order of the polynomial used to fit the vocal fold geometry. A performance of the analysis is relatively insensitive to the threshold value in the edge detection, in part due to the averaging employed in those steps, as well as the polynomial fit performed on the extracted points. On the other hand, proper identification of the VF attachment points is critical for a good kinematic representation of the VFs, with inaccurate identification of these points resulting in poor representations of VF trajectories, which, in turn, affects pressure estimations. Higher order polynomials (e.g., *p* = 3 or 4) yielded poor and unstable results in the CPA estimation. Good and smooth fits in the registration stage were correctly obtained, but the VF trajectories during the collision phase missed the cinematic coherence. We attribute this issue to the KF model for higher order coefficients in Θ_*k*_. In these cases, the variability of each *θ*_*i,k*_ is not well-represented with the simple harmonic motion. As such, while the presented framework is derived to enable higher-order polynomial representations, from a practical perspective, we recommend lower-order (i.e., quadratic) fits for optimal performance.

Future efforts will consider additional silicone model validation under more controlled lighting conditions and additional model configurations (multiple elastic moduli and subglottal pressures) as well as in vivo validation where contrast with VF contact pressure is possible. Other avenues of research include exploring this technique in a large pool of subjects, including patients with voice disorders (e.g., nodules and polyps) and adopting the approach for stroboscopic acquisition methods.

## 6. Conclusions

Current preliminary results of the collision pressure estimations are in good agreement with both silicone vocal fold model experiments and previous studies. This indicates that the KF edge tracking with a Hertz collision model is feasible for the purpose of obtaining simple estimates of contact pressure using solely kinematic vocal fold information. The method is noninvasive and does not need probes, controlled lab conditions, or speckle patterns. The method does not require previous knowledge about VF tissue parameters and does not require calibrated videos in physical units. The proposed scheme simplifies the contact estimation problem, minimizes estimation bias, and is ideal for studying same-subject variations. Thus, the method is promising for enhancing the objective assessment of vocal function in clinical settings, especially considering its potential usability in stroboscopic systems.

## Figures and Tables

**Table 1.** Material properties of the synthetic vocal fold models.

Layer	Modulus of Elasticity kPa
Adipose tissue	6.8
Body	17.3
Cover	0.95–2.45
Epithelium	101.0

**Table 2.** Contact Pressure Analysis (CPA) parameters for synthetic and in vivo high-speed videoendoscopy (HSV) material.

Parameter	Symbol	Synthetic	In Vivo HSV	Unit
Sampling rate	*f*_*s*_	8750	8000	(fps)
Young modulus	*E*	24.75	24.75	(kPa)
Poisson ratio	*ν*	0.5	0.5	-
Polynomial order	*p*	2	2	-
Gradient threshold	*t*_*h*_	0.5	0.25	-
Beta	*β*	20	20	-
Gamma	*γ*	0.5	0.5	-

**Table 3.** CPA results for a silicone model validation for a cover with modulus of elasticity, *E*_*cover*_ = 1.65 kPa. The results are mean values from more than 30 cycles of peak contact.

Subglottal Pressure kPa	Contact Pressure (Measured from Probe) kPa	4 *α · δ*_*c*_*/L*_*c*_ (Measured from HSV)	Equivalent Elasticity E* (Estimated) kPa
2.2	2.2	0.102	22.0
2.4	2.6	0.332	7.9
2.6	3.3	0.528	6.2
2.8	3.5	0.262	13.2
3.0	3.7	-	-
3.2	3.9	0.166	23.6
3.4	4.3	0.244	17.6

**Table 4.** CPA results for silicone model validation. The measured contact pressure is contrasted with the estimated contact pressure. The mean value and standard deviation are reported for the latter.

Cover Young’s	Subglottal Pressure	Contact Pressure	Contact Pressure
Modulus kPa	(Static) kPa	(Measured) kPa	(Estimated) kPa
0.95	2.15	2.47	1.93 ± 0.92
2.45	2.20	4.84	4.70 ± 2.25

**Table 5.** CPA results for in vivo HSV case of study. The CPA estimates include the absolute pressure and the relative pressure with respect to the normal loudness case.

Subjective Loudness	Sound Pressure Level dB ref: 20 μPa	Contact Pressure (Estimated) kPa	Ratio with Respect to *Normal Loudness*
*Soft*	72.9	1.03	0.58
*Normal*	81.8	1.77	1.00
*Loud*	93.3	3.76	2.13
